# The PhysioFlow Thoracic Impedancemeter Is Not Valid for the Measurements of Cardiac Hemodynamic Parameters in Chronic Anemic Patients

**DOI:** 10.1371/journal.pone.0079086

**Published:** 2013-10-22

**Authors:** Pascal Bogui, Edwige Balayssac-Siransy, Philippe Connes, Nalourgo Tuo, Soualiho Ouattara, Aurélien Pichon, Cyrille Serges Dah

**Affiliations:** 1 Laboratoire de Physiologie et d’Explorations Fonctionnelles, Unité de Formation et de Recherche en Sciences Médicales, Université Félix Houphouët Boigny, Abidjan, Côte d’Ivoire; 2 Service des explorations fonctionnelles et endoscopiques, Centre hospitalier universitaire de Yopougon, Abidjan, Côte d’Ivoire; 3 UMR Inserm 665, Université des Antilles et de la Guyane, Pointe-à-Pitre, Guadeloupe; 4 Laboratoire ACTES (EA 3596), Département de Physiologie, Université des Antilles et de la Guyane, Pointe-à-Pitre, Guadeloupe; 5 Laboratory of Excellence GR-Ex « The red cell: from genesis to death », PRES Sorbonne, Paris, France; 6 Laboratoire «Réponses cellulaires et fonctionnelles à l'hypoxie» EA2363, Université Paris 13 - PRES Sorbonne Paris Cité, Bobigny, France; 7 Service des explorations fonctionnelles respiratoires, Centre hospitalier universitaire de Cocody, Abidjan, Côte d’Ivoire; Royal College of Surgeons, Ireland

## Abstract

The aim of the present study was to test the validity of the transthoracic electrical bioimpedance method PhysioFlow® to measure stroke volume in patients with chronic anemia. Stroke volume index (SVI), as well as cardiac index (CI) obtained by transthoracic electrical bioimpedance method and doppler echocardiography were compared in healthy subjects (n = 25) and patients with chronic anemia (i.e. mainly with sickle cell anemia; n = 32), at rest. While doppler echocardiography was able to detect difference in SVI between the two populations, the Physioflow® failed to detect any difference. Bland & Altman analyses have demonstrated no interchangeability between the two methods to assess CI and SVI in anemic patients and healthy subjects. While doppler echocardiography displayed a good concordance for SVI results with those obtained in the literature for anemic patients, the Physioflow® did not. Finally, in contrast to doppler echocardiography: 1) the CI obtained with the Physioflow® was not correlated with the hemoglobin level and 2) the stroke volume determined by the Physioflow® was highly influenced by body surface area. In conclusion, our findings indicate that the Physioflow® device is inaccurate for the measurement of SVI and CI in patients with chronic anemia and has a poor accuracy for the measurement of these parameters in African healthy subjects.

## Introduction

While echocardiography techniques are easily available in high-income economies countries for measuring cardiac function and hemodynamic parameters, they are less accessible and expensive for developing countries. The use of alternate non-invasive techniques such as transthoracic electrical bioimpedance methods (PhysioFlow®) are thus of great interests in developing countries because of the limited economic cost and the facility of use. A growing interest has been devoted to the PhysioFlow® within the past decades. However, very few studies performed on small number of sportsmen or patients with lung diseases have suggested that the PhysioFlow® could provide a clinically acceptable and non-invasive evaluation of the cardiac output, at rest and during exercise, compared to the direct Fick, CO_2_ rebreathing or the indocyanine-green dye dilution methods [1–4]. In contrast, some studies challenged the accuracy of the PhysioFlow® to measure cardiac function in healthy volunteers with dopamine infusion [5], in anesthetized children with congenital heart disease [6], under challenging situation such as fluid replacement, blood transfusion or hemodialysis [7] and in pediatric patients with and without cardiac disease undergoing anesthesia for magnetic resonance imaging [8]. Indeed, the validity of the PhysioFlow® and its accuracy are still not clearly established. 

Chronic anemic disorders are frequent in developing areas, such as in Africa. Patients with sickle cell anemia (SCA) and other chronic anemic disorders have increased cardiac output (Qc) [9–11] resulting mainly from a large rise of stroke volume (SV) [10–17] and a slight increase of heart rate at steady state [18,19]. During acute hemolytic episodes or painful vaso-occlusion (for SCA), the acute decrease of hemoglobin results in a rise of cardiac output, which may reach the limits of cardiovascular adaptation, hence increasing the risks for heart failure [20]. Thus, the determination of cardiac function and hemodynamic adaptations in patients with chronic anemia is of specific interest to assess the clinical severity and the risks for cardiovascular complications. 

The aim of the present study was therefore to test the validity of the PhysioFlow® to measure the stroke volume in patients with chronic anemia. Stroke volumes obtained by transthoracic electrical bioimpedance method were compared to those acquired with doppler echocardiography in healthy subjects and patients with chronic anemia (mainly SCA). 

## Materials and Methods

### Patients

The study took place at the Academic Hospital of Yopougon (Abidjan, Ivory Coast), and included 32 adults with chronic anemia (30 with SCA, 1 with both normal hemoglobin and hemoglobin C, 1 with normal hemoglobin) and 25 healthy adults. Subjects with cardiovascular or pulmonary diseases, or smoking habits were excluded from the study. All patients were in steady-state condition at the time of the study: no blood transfusions in the previous three months and absence of acute episodes (infection, vaso-occlusive events, stroke, priapism) at least two months before inclusion into the study. The study was conducted in accordance with the guidelines set by the Declaration of Helsinki and was approved by the Ethics Committee of the Academic Hospital of Yopougon (Abidjan, Ivory Coast). All patients were advised about the purpose and procedures of the study, and gave their informed written consent. 

### Stroke volume measurements

Stroke volume (SV) was determined for each subject simultaneously by transthoracic electrical bioimpedancemeter (PhysioFlow®, Manatec type PF05L1, Paris, France) and doppler echocardiography (Hewlett Packard, Sonos 2000, USA), which was considered as the gold standard, after 30 minutes of rest in supine position. Since no specific position is recommended for the hemodynamics measurement by the PhysioFlow®, the position adopted by the subject was the one recommended for doppler echocardiographic analyses: left lateral decubitus with left hand placed under the head and chest free. 

For the Physioflow®, after gently skin scraping and as recommended by the manufacturer’s instructions, six electrodes (Skintact FS-50) were used: 2 on the neck, 2 at the xiphisternum and 1 on each side of the chest. The bioimpedance method uses changes in transthoracic impedance during cardiac ejection to calculate SV [1,2,4]. Heart rate (HR) determination is based on the R-R interval duration determined using the first derivative of the electrocardiogram (ECG). Adequate signal quality for interpretation was detected by a color graph. Artifact detection was diagnosed by the PhysioFlow® device and later by one of the investigators (PB) who performed manual review of the data and identified data thought to be “physiologically implausible”: i.e. when measuring, for a given subject, SV over time, values greater than the mean SV +/-20% (if HR was unchanged; i.e. mean +/-5%) were deleted from the series. After an initial calibration method of 20 seconds, continuous hemodynamic measurements were performed for 25 minutes. Values at the 15^th^ and 20^th^ minute (10 seconds recording) were averaged. When the two measurements were not reproducible (difference > 5%), a third measure was done at the 25^th^ min and averaged with the two preceding values. The Physioflow designers did not reveal the exact formula for hemodynamical measurements and SV calculations, and only little information is available in a previous study [1]. The starting of the PhysioFlow® software program requires the recording of information: sex, age, height, weight, and systolic and diastolic blood pressures.

Doppler echocardiographic measurements were performed at the 15^th^ and 20^th^ min (and 25^th^ min if needed), and averaged, using echocardiography device with 5 MHz transducer. The stroke volume was calculated by the product of aortic annular plane and velocity time integral of aortic flow, as recommended [21,22]. The value used is the mean of 5 measures. After heart rate (HR) measurement, cardiac output was calculated (CO = SV * HR). 

The cardiac index (CI = CO/body surface area) and the stroke volume index (SVI = SV/body surface area) were calculated for each subject and each method.

#### Statistics

Values are expressed as means ± standard deviation (SD). Significance level was defined as p < 0.05. Analyses were conducted using SPSS (v. 20, IBM SPSS Statistics, Chicago, IL).

#### Reproducibility of the two methods

The reproducibility of each method to measure SVI has been studied in 10 healthy subjects during 4 consecutive days. Anthropometric and blood pressures remained unchanged during this period. The coefficient of variation (CV) of each method was calculated from the average of the CV determined in the 10 healthy subjects over the 4 experimental days. A two-way analysis of variance (ANOVA) with repeated measures was used to compare the values of SVI between the two methods over the 4-days period. An unpaired t test was used to compare the mean CV between the two methods. 

#### Comparison of the two methods

The hemodynamic measurements were compared between the two groups, and between the two methods, using an unpaired Student t test. A Pearson test was used to assess the correlation between the CI and SVI values obtained by the two methods. 

The interchangeability between the two methods (CI and SVI parameters) was evaluated by the Bland-Altman analysis [23]. To test whether the mean difference between the two methods was not significantly different from 0, a one-sample Student t test procedure was performed. Finally, a linear regression was drawn on the Bland-Altman scatter plot to assess possible changes in bias with the increase in CI or SVI. The acceptable limit of agreement to assess the interchangeability between the two methods of hemodynamic measurements was fixed at 30% of the mean values [24,25]. Above this limit, the physiological and clinical meanings of the measurement could be considered doubtful. The Bland-Altman analysis was applied in each group (healthy and anemic) and in all subjects.

#### Physiological correlates of the two methods

Pearson test was performed between the hemoglobin level and the values of CI or SVI obtained with each method. Finally, the impact of fictitious changes in body surface area (BSA) on SV measurement was assessed for the two methods in one representative subject. The fictitious values of height and weight were entered manually into the softwares of the PhysioFlow® and echocardiography device before measurement, to obtain, according to the Dubois’s formula (CATO Software Solutions), fictitious body surface areas (BSA) of 1.50 m^2^; 1.60 m^2^; 1.70 m^2^; 1.80 m^2^; 1.90 m^2^; 2.00 m^2^; 2.10 m^2^ and 2.20 m^2^. These values lead to normal body mass indices between 22 and 25 Kg/m^2^ and close to the real one of the tested subject. The values were randomly assigned and entered into the software of both devices and the measurements of SV and SVI were realized.

#### Comparison of the two methods with previous studies

A Student ‘t’ test was used to compare the CI and SVI values from the present study with the values obtained in previous studies on healthy subjects and on chronic anemic disorders. The studies selected for comparisons were those where the values (i.e., sample size, means and SD) were available.

## Results

The characteristics of the subjects are reported in the table 1. 

**Table 1:** Characteristics of the two groups.

	**Sex ratio (H/F)**	**Age (yrs)**	**Height (cm)**	**Weight (kg)**	**Body surface area (m^2^)**	**Hb level (g/dL)**	**HR (bpm)**	**Systolic blood pressure (mmHg)**	**Diastolic blood pressure (mmHg)**
**Healthy subjects (n=25)**	25/0	24 ± 3	174 ± 5	65 ± 6	1.78 ± 0.10	14.4 ± 1.3	67 ± 7	121 ± 9	76 ± 8
**Anemic patients (n=32)**	28/4	26 ± 5	169 ± 10^$^	56 ± 10^$$$^	1.61 ± 0.18^$$$^	8.6 ± 1.4^$$$^	74 ± 11^$$^	116 ± 10	67 ± 9^$$$^

Means ± SD. Hb = Hemoglobin; HR = heart rate. Difference between healthy subjects and chronic anemic patients (^$^p<0.05, ^$$^p<0.01; ^$$$^p < 0.001).

### Reproducibility of the two methods

The mean reproducibility of SVI over the 4-days period in 10 healthy subjects was not significantly different between doppler echocardiography and PhysioFlow® methods (Table 2). The mean SVI values did not change with time and the data obtained with the two methods were not different.

**Table 2:** Mean values of SVI in 10 healthy subjects measured during 4 consecutive days by doppler echocardiography and PhysioFlow® methods.

	**SVI (mL/m^2^)**				
	**D1**	**D2**	**D3**	**D4**	**CV (%)**
**Doppler Echocardiography**	42 ± 5	44 ± 5	46 ± 6	44 ± 5	8.4 ± 3.8
**PhysioFlow**®	44 ± 5	46 ± 5	49 ± 6	47 ± 5	5.7 ± 2.2

Means ± SD. SVI = stroke volume index; D1, D2, D3 and D4 = day1, 2, 3 and 4; CV = coefficient of variation.

### Comparison of the two methods

As expected, using doppler echocardiography, patients with chronic anemia had greater CI (p < 0.01) and SVI (p < 0.001) than the group of healthy subjects (Table 3). In contrast, while CI remained significantly different between the two groups using the PhysioFlow® (p < 0.01), SVI did not remain statistically different. In the healthy group, the CI and SVI values did not significantly differ between the two methods (Table 3). In contrast, both CI and SVI values (p < 0.001) were lowered in the anemic group with the PhysioFlow® as compared to doppler echocardiography.

**Table 3:** Mean CI and SVI values measured by doppler echocardiograpgy and PhysioFlow® methods in healthy subjects and patients with chronic anemia.

	**CI (L/min/m^2^)**		**SVI (mL/m^2^)**	
	**Doppler Echocardiography**	**PhysioFlow**	**Doppler Echocardiography**	**PhysioFlow**
**Healthy subjects (n=25)**	3.10 ± 0.42	2.98 ± 0.62	47 ± 6	44 ± 6
**Anemic patients (n=32)**	4.90 ± 0.99 ^$$^	3.41 ± 0.76*** ^$^	68 ± 12^$$$^	47 ± 9***

Means ± SD. CI = cardiac index; SVI = stroke volume index. Difference between doppler echocardiography and PhysioFlow (***p < 0.001); difference between healthy subjects and chronic anemic patients (^$^p<0.05, ^$$^p<0.01; ^$$$^p < 0.001).

### Correlation between the two methods

**Figure 1:**
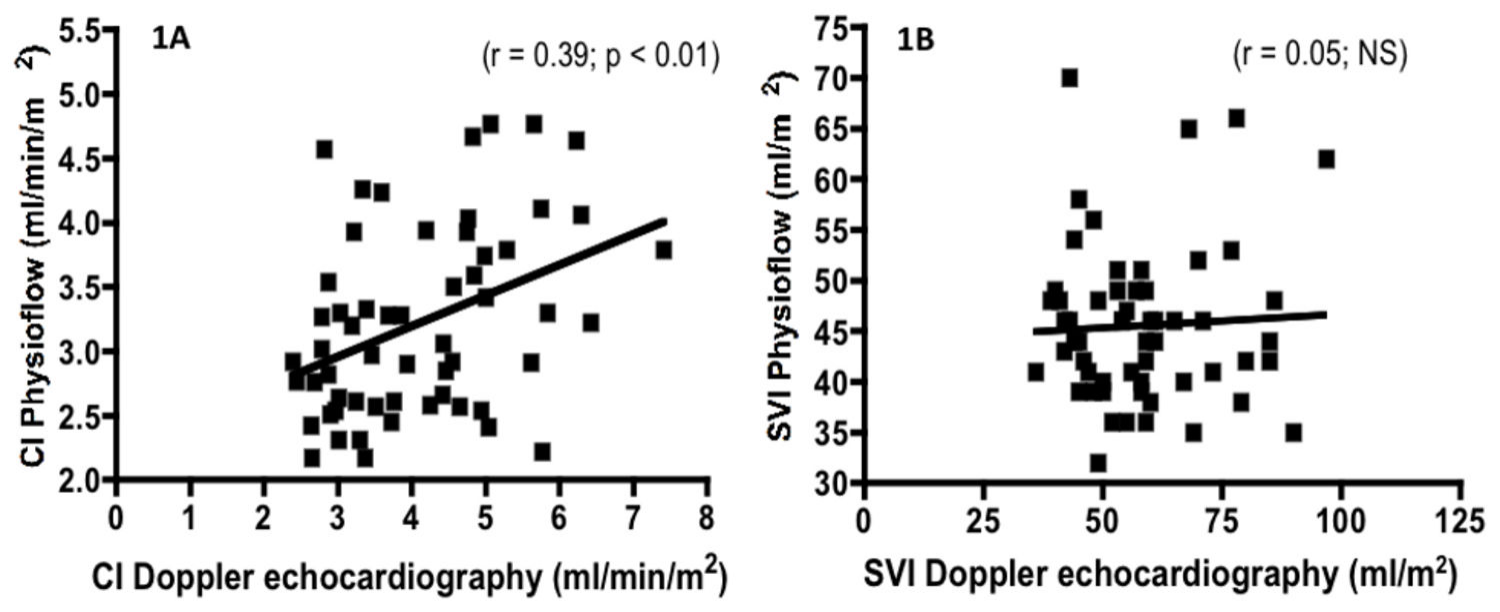
Correlation between CI (1A) or SVI (1B) measured by doppler echocardiography and determined by the PhysioFlow®.

Pearson analysis demonstrated no correlation between SVI determined by doppler echocardiography and SVI determined by the PhysioFlow® (Figure 1B). Moreover, a positive, but weak, correlation was found between CI determined by Doppler echocardiography and CI determined by the PhysioFlow® (r = 0.39; p < 0.01; Figure 1A). 

### Bland and Altman analysis

**Figure 2:**
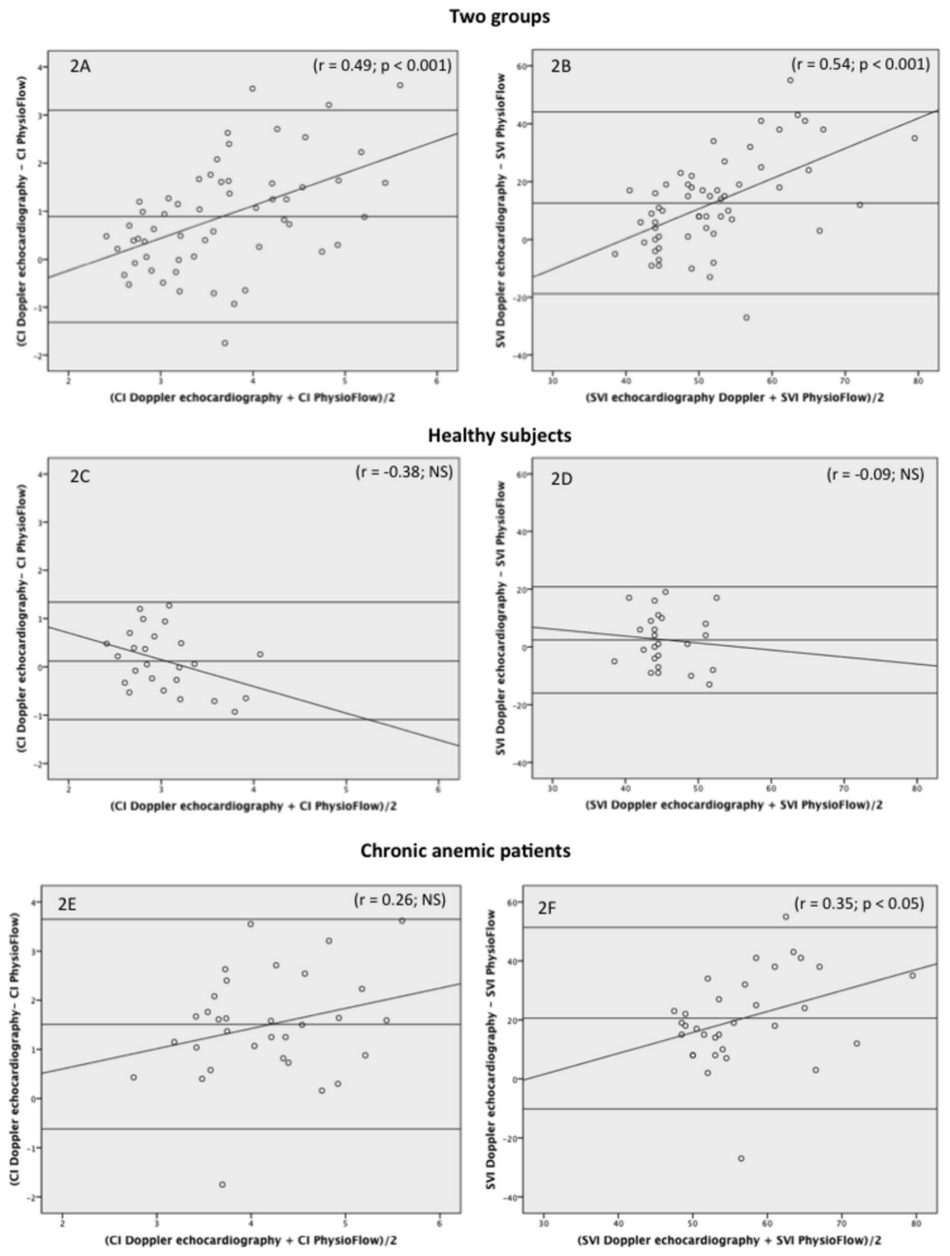
Bland and Altman analysis on CI and SVI determined by the two methods in the whole group (2A and 2B), the healthy subjects (2C and 2D) and the chronic anemic patients (2E and 2F).

The results of the Bland and Altman analysis are displayed in Figure 2 and Table 4. 

**Table 4:** Bland & Altman analysis results for assessment of intercheangeability between doppler echocardiograpgy and PhysioFlow® methods in healthy subjects, patients with chronic anemia and both groups.

		**Bias** (95%CI)	**Bias vs 0** (P values)	**Lower limits of agreement** (95%CI)	**Upper limits of agreement** (95%CI)	**Limit of Agreement** (% mean values)
**Healthy subjects (n=25)**	*Cardiac index*	0.1 (-0.1;0.4)	NS	-1.1 (-1.5;-0.6)	1.3 (0.9;1.8)	40%
	*SV index*	2.4 (-1.5;6.3)	NS	-16 (-22.7;-9.3)	20.8 (14.1;27.5)	40%
**Anemic patients (n=32)**	*Cardiac index*	1.5 (1.1;1.9)	***	-0.6 (-1.3;0.1)	3.6 (2.9;4.3)	54%
	*SV index*	20.6 (14.9;26.3)	***	-10.2 (-20.0;-0.4)	51.4 (41.6;61.2)	54%
**All (n=57)**	*Cardiac index*	0.9 (0.6;1.2)	***	-1.3 (-1.8;-0.8)	3.1 (2.6;3.6)	60%
	*SV index*	12.6 (8.4;18.9)	***	-18.8 (-26.2;-11.5)	44.1 (36.7;51.4)	60%

Means ± SD. NS = non significant; statistically different from 0 (***p < 0.001).

The Figures 2A and 2B show the results in the whole group (healthy subjects + anemic patients) for the Bland and Altman analysis on CI and SVI, respectively. For both CI and SVI, the bias was significantly different from 0 (p < 0.001) suggesting a mean difference between the two methods. The significant positive linear regression shows a tendency for the bias to become positive with increasing CI (r = 0.49; p < 0.001) or increasing SVI (r = 0.54; p < 0.001). The limit of agreement represents 60% (> fixed limit of agreement) of the mean values of CI or SVI, suggesting no interchangeability between the two methods. 

The Figures 2C and 2D show the results in the healthy group for the Bland and Altman analysis on CI and SVI, respectively. The limit of agreement represents 40% (> fixed limit of agreement) of the mean values of CI or SVI, suggesting no interchangeability between the two methods.

The Figures 2E and 2F show the results in the chronic anemic group for the Bland and Altman analysis on CI and SVI, respectively. For both CI and SVI, the bias was significantly different from 0 (p < 0.001) suggesting a mean difference between the two methods. While the linear regression for SVI was not significant, the significant positive linear regression for SVI shows a tendency for the bias to become positive with increasing SVI (r = 0.35; p < 0.05). The limit of agreement represents 54% (> fixed limit of agreement) of the mean values of CI or SVI, suggesting no interchangeability between the two methods. 

### Physiological correlates of the two methods

**Figure 3:**
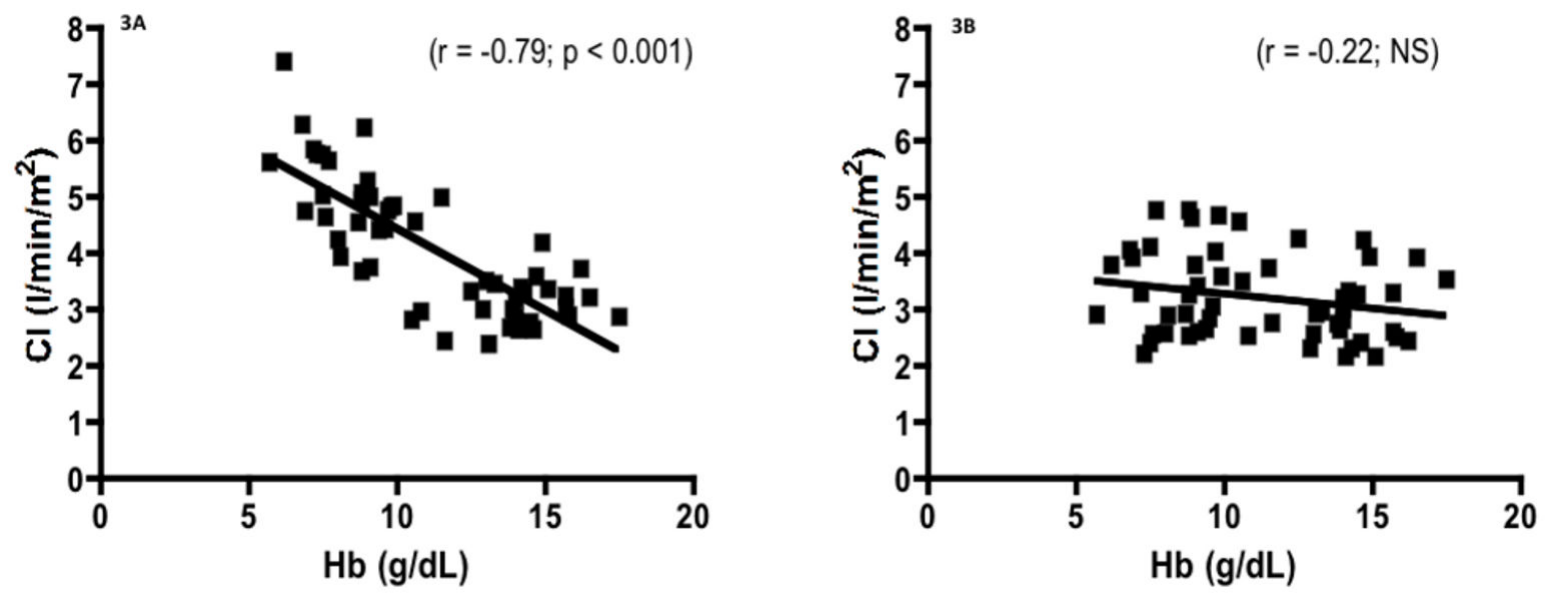
Correlation between CI measured by doppler echocardiography (a) or determined by the PhysioFlow® (3B) and the hemoglobin (Hb) level.

**Figure 4:**
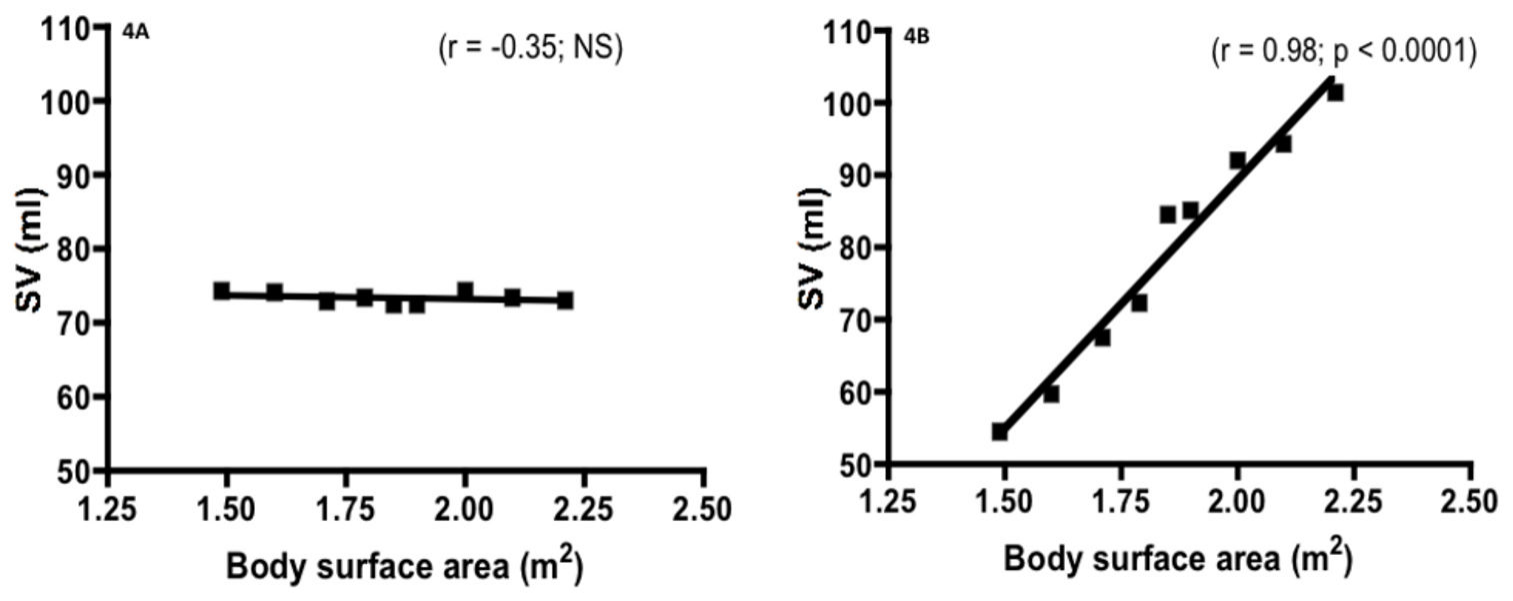
Effects of fictitious body surface area values on stroke volume (SV) measured by doppler echocardiography (4A) or by the PhysioFlow® (4B) in a given subject.

The Figure 3 shows the correlation between CI measured either by doppler echocardiography (3A) or by the PhysioFlow (3B) and the haemoglobin level in all subjects. While the relationship was significant and negative when CI was determined by doppler echocardiography, the correlation was not significant when CI was measured by the PhysioFlow method. The Figure 4 shows the effects of fictitious BSA values on SV measurements by doppler echocardiography (4A) or by the PhysioFlow® (4B) in a given subject. While SV determined by doppler echocardiography was not affected by the fictitious values of BSA, we reported a significant positive linear relationship between SV measured by the PhysioFlow® and BSA. We also found a significant negative correlation between fictitious BSA and SVI values with echocardiography (r = -0.99; p < 0.0001): this is a logical result since the unchanged SV (whatever the BSA values manually entered) was divided by an increasing artificial BSA. However, by using the Physioflow®, we found a significant and very surprising positive correlation between SVI and BSA (r=0.91; p < 0.001). 

### Comparison of the two methods with previous selected studies

The table 5 shows the results of the comparisons between the mean CI or SVI values obtained in the present study with both methods and the CI or SVI values obtained with various methods in healthy subjects in previous studies. No difference was observed for CI. Regarding SVI, one study [26] reported slightly higher values than those observed in the present study with both methods and another one [27] found slightly lower values than our results obtained with doppler echocardiography. The values from the present study, whatever the methods used for the determination of CI and SVI, are very closed to the values published in the literature.

**Table 5:** Comparisons between the CI and SVI values obtained in the present study with doppler echocardiography or PhysioFlow® and the values obtained in previous selected studies on healthy subjects.

**Study**	**Method used**	**CI (l/min/m^2^)**	**Comparison with the present study**		**SVI (ml/m^2^)**	**Comparison with the present study**	
			**Doppler echocardiography**	**PhysioFlow**		**Doppler echocardiography**	**PhysioFlow**
Present study	Doppler echocardiography (n=25)	3.1 ± 0.4			47 ± 6		
	Physioflow (n=25)	3.0 ± 0.6			44 ± 6		
[26]	Indicator-dilution method (n=4)	3.5 ± 0.2	NS	NS	54 ± 2	*	*
[31]	Indicator-dilution method (n=4)	3.1 ± 0.4	NS	NS	50 ± 9	NS	NS
[32]	Fick method (n=11)	3.2 ± 0.2	NS	NS	43 ± 2	NS	NS
[13]	Fick method (n=7)	3.2 ± 0.4	NS	NS	43 ± 5	NS	NS
[33]	Bidimensional echocardiography (n=9)	-	-	-	47 ± 2	NS	NS
[34]	Indicator-dilution method (n=4)	3.5 ± 0.3	NS	NS	50 ± 5	NS	NS
[16]	Fick method (n=6)	3.2 ± 0.3	NS	NS	46 ± 9	NS	NS
[35]	Bidimensional echocardiography (n=11)	2.8 ± 0.8	NS	NS	-	-	-
[27]	Bidimensional echocardiography (n=7)	-	-	-	42 ± 4	*	NS
[36]	Doppler echocardiography (n=30)	3.6 ± 1.1	NS	NS	-	-	-
[37]	Doppler echocardiography (n=25)	3.1 ± 0.9	NS	NS	-	-	-
[38]	Resonnance Magnetic Imaging (n=76)	-	-	-	45 ± 9	NS	NS

Means ± SD. NS = non significant; statistical difference between the present study and previous study (*p < 0.05).

The table 6 shows the results of the comparisons between the mean CI or SVI values obtained in the present study with both methods and the CI or SVI values obtained in patients with sickle cell anemia in previous studies. While CI determined by echocardiography in our study differed from only one published study, CI determined by the Physioflow® was significantly different from 7 of the 9 studies presented. SVI determined by echocardiography in our study was not different from other studies. In contrast, 3 of the 5 studies presented reported SVI values statistically different from SVI values determined by the Physioflow® in the present study. 

**Table 6:** Comparisons between the CI and SVI values obtained in the present study with doppler echocardiography or PhysioFlow® and the values obtained in previous selected studies in patients with sickle cell anemia.

**Study**	**Method used**	**Hb level (g/dL)**	**CI (l/min/m^2^)**	**Comparison with the present study**		**SVI (ml/m^2^)**	**Comparison with the present study**	
				**Doppler echocardiography**	**PhysioFlow**		**Doppler echocardiography**	**PhysioFlow**
Present study	Doppler echocardiography (n=32)	8.6 ± 1.4	4.9 ± 1.0			67 ± 13		
	Physioflow (n=32)	8.6 ± 1.4	3.4 ± 0.8			47 ± 9		
[30]	Fick method (n=12)	7.9 ± 1.6	6.0 ± 0.8	**	***	-	-	-
[10]	Fick method (n=9)	9.6 ± 2.0	4.2 ± 0.4	NS	NS	-	-	-
[39]	Fick method (n=7)	7.5 ± 1.1	7.3 ± 2.6	NS	NS	77 ± 26	NS	NS
[33]	Bidimensional echocardiography (n=23)	-	-	-	-	64 ± 14	NS	*
[16]	Fick method (n=39)	8.2 ± 1.4	5.6 ± 0.2	NS	***	69 ± 14	NS	***
[40]	Cardiac scintigraphy (n=10)	7.9 ± 1.1	4.9 ± 1.2	NS	***	69 ± 20	NS	NS
[35]	Bidimensional echocardiography (n=11)	7.9 ± ?	4.7 ± 1.1	NS	***	-	-	-
[41]	Doppler echocardiography (n=10)	7.8 ± 0.6	5.5 ± 1.3	NS	***	61 ± ?	-	-
[27]	Bidimensional echocardiography (n=13)	6.7 ± 1.0	-	-	-	58 ± 5	NS	*
[36]	Doppler echocardiography (n=40)	8.9 ±1.7	4.8 ± 0.4	NS	***	-	-	-
[37]	Doppler echocardiography (n=25)	-	5.9 ± 1.8	NS	*	_	-	-

Means ± SD. ? = not communicated by the authors; NS = non significant; statistical difference between the present study and previous study (*p < 0.05; **p < 0.01; ***p < 0.001).

## Discussion

Our study is the first to describe the performance characteristics of an electrical impedance monitor (Physioflow® device) in a group of healthy African and in a group of African patients with chronic anemia (mainly sickle cell anemia). Our main findings demonstrated an inability of the Physioflow® to detect any difference in SVI between the two populations while standard doppler echocardiography was accurate. Moreover, there was no interchangeability according to the Bland & Altman and correlations analyses between the Physioflow® device and standard doppler echocardiography for the measurements of CI and SVI in anemic patients and healthy subjects. Whereas the standard doppler echocardiography displayed a good concordance for CI and SVI results with those obtained in the literature in anemic patients, the Physioflow did not. Finally, in contrast to standard doppler echocardiography: 1) the CI measured by the Physioflow was not correlated with the hemoglobin level and 2) the stroke volume determined with the Physioflow was highly correlated with BSA. On the whole, our findings indicate that the Physioflow® device is inaccurate for the measurement of SVI and CI in patients with chronic anemia and has a poor accuracy for the measurement of these parameters in African healthy subjects.

The Physioflow® used in this study is one of the “second-generation” devices using bioimpedance technology to determine cardiac hemodynamic parameters. Historically, the clinical use of bioimpedance methods (i.e. “first-generation devices”) to measure cardiac hemodynamic parameters was hampered by baseline instability. Indeed, the development of the Physioflow® has included a modified proprietary algorithm, which avoids calculation of baseline impedance. The basic equation for calculating stroke volume has been modified, avoiding the calculation of blood resistivity, the distance between the recording electrodes and the Z0 used in the original equations from Kubicek and Sramek-Bernstein [1,6]. 

The validity and accuracy of the Physioflow® has been recently challenged in pediatrics population with and without cardiac diseases [6,8]. The authors reported a significant but very weak correlation between CI measured by mass spectrometry and Physioflow® and a poor agreement between the two methods [6,8]. It was demonstrated that the Physioflow® device significantly overestimated CI in patients with biventricular physiology and underestimated it in children with univentricular physiology [6]. When the Physioflow® was compared with the Fick method in children with cardiac disease, the mean bias of CI was -0.09 l/min, but the 95% limits of agreement were -3.30 to +3.01 l/min [6]. Only 20 of 56 (36%) measurements were within 20%, and 31 of 56 (55%) of measurements were within 30% of each other [6]. 

Our results support a lack of accuracy of the Physioflow® device to measure CI and SVI in African patients with chronic anemia. We found a poor agreement with doppler echocardiography and no interchangeability between the two devices. In addition, while the values of SVI and CI obtained with doppler echocardiography in chronic anemic patients are close to the ones published in the literature using various standard methods (table 6), large differences have been found with the Physioflow® device. Increased CI and SVI in chronic anemic patients, and particularly in sickle cell anemia [16], is an adaptation to the anemia in order to maintain adequate tissues oxygen supply [16,28]. The decreased cardiac preload and afterload caused by the lower hemoglobin level and blood viscosity [29] are known to be responsible for the increased stroke volume in chronic anemic patients [11] and are at the origin of significant dilation of cardiac chambers in this population [19]. While the use of doppler echocardiography was able to detect difference in SVI between healthy group and the chronic anemic group, the Physioflow® failed to detect any difference. Moreover, SVI values measured by the two methods were not correlated. The relationship found in the present study between CI measured by doppler echocardiography and hemoglobin level is a classical observation published several times by other groups [11,30]. But, surprisingly and in contrast with these studies, no association was found between the hemoglobin level and CI measured by the Physioflow®. These findings add doubt about the ability of the Physioflow® to determine cardiac hemodynamic parameters, particularly in chronic anemic patients. Although the results obtained in the healthy group support a greater accuracy of the Physioflow® to measure cardiac hemodynamic parameters in healthy population than in chronic anemic patients, the poor interchangeability with doppler echocardiography demonstrates that Physioflow® can not be used with a high confidence in the general population. We are unable to explain why the Physioflow® lacks of accuracy for the measurements of cardiac hemodynamic parameters, mainly SVI, because the algorithms are not fully available [1]. Finally, we reported a strong relationship between body surface area and stroke volume, which is not the case with doppler echocardiography. These results indicate that height and weight are probably key parameters in the algorithm used by the Physioflow® to determine hemodynamical parameters, which is not the case with doppler echocardiography. 

In conclusion, the accuracy of Physioflow® using its current algorithms does not support its use for clinical monitoring of cardiac hemodynamic parameters in chronic anemic patients. In addition, caution is required regarding its use in the general population due to the large influence of the anthropometric data in the SV calculation. 
